# The Curious Case of Benzbromarone: Insight into Super-Inhibition of Cytochrome P450

**DOI:** 10.1371/journal.pone.0089967

**Published:** 2014-03-03

**Authors:** Abhinav Parashar, Sudeep Kumar Gade, Mahesh Potnuru, Nandita Madhavan, Kelath Murali Manoj

**Affiliations:** 1 REDOx Lab, Biotechnology and Healthcare Division, PSG Institute of Advanced Studies, Peelamedu, Tamil Nadu, India; 2 School of Biosciences and Technology, VIT University, Vellore, Tamil Nadu, India; 3 Department of Chemistry, IIT Madras, Chennai, Tamil Nadu, India; Concordia University Wisconsin, United States of America

## Abstract

Cytochrome P450 (CYP) family of redox enzymes metabolize drugs and xenobiotics in liver microsomes. Isozyme CYP2C9 is reported to be inhibited by benzbromarone (BzBr) and this phenomenon was hitherto explained by classical active-site binding. Theoretically, it was impossible to envisage the experimentally derived sub-nM K_i_ for an inhibitor, when supra-nM enzyme and 10X K_M_ substrate concentrations were employed. We set out to find a more plausible explanation for this highly intriguing “super-inhibition” phenomenon. *In silico* docking of various BzBr analogs with known crystal structure of CYP2C9 did not provide any evidence in support of active-site based inhibition hypothesis. Experiments tested the effects of BzBr and nine analogs on CYPs in reconstituted systems of lab-purified proteins, complex baculosomes & crude microsomal preparations. In certain setups, BzBr and its analogs could even enhance reactions, which cannot be explained by an active site hypothesis. Generally, it was seen that K_i_ became smaller by orders of magnitude, upon increasing the dilution order of BzBr analogs. Also, it was seen that BzBr could also inhibit other CYP isozymes like CYP3A4, CYP2D6 and CYP2E1. Further, amphipathic derivatives of vitamins C & E (scavengers of diffusible reactive oxygen species or DROS) effectively inhibited CYP2C9 reactions in different reaction setups. Therefore, the inhibition of CYP activity by BzBr analogs (which are also surface-active redox agents) is attributed to catalytic scavenging of DROS at phospholipid interface. The current work expands the scope of interpretations of inhibitions in redox enzymes and ushers in a new cellular biochemistry paradigm that small amounts of DROS may be obligatorily required in routine redox metabolism for constructive catalytic roles.

## Introduction

Cytochrome P450 (CYP) family of enzymes mediate the metabolism of a majority of xenobiotics in mammals. In man, CYP2C9 is responsible for ∼20% of phase I drug metabolism, particularly of the non-steroidal anti-inflammatory agents (NSAIDs) possessing an anionic moiety [1], [2]. It was found that while the crystallized chimera/mutant of CYP2C9 (1OG5) had no positively charged residues within the active site, Arg108 was a probable candidate for binding of anionic moieties in the active site of CYP2C9 (1R9O) [1], [3]. Therefore, it was argued that the 1R9O structure better explained the catalytic activities of CYP2C9. Among the inhibitors reported for CYPs, benzbromarone (BzBr) and its iodinated analogue, benziodarone (BzIr) have been suggested to be the most potent for CYP2C9, with K_i_ value of ≤1 nM [4]–[6]. One of us found that the values of inhibition constants derived from experimental rate measurements ‘stretched’ the intuitive understanding afforded by the theoretical model. This is because the practical enzyme inhibition assays are performed with tens to hundreds of nM CYPs and ∼100 µM concentration of substrates (at ∼10X K_M_). Therefore, several analogues/derivatives of benzbromarone were employed to analyze the veracity of assumptions made, checking the inhibition profiles in a wide array of CYPs' reaction setups. The data thus obtained was analyzed in many modalities. Along with the *in vitro* approach, *in silico* docking was also probed for evidence to support the hitherto available model. The work provides an alternative explanation for the inhibitions observed and gives key insights into the mechanistic aspects of cytochrome P450 mediated redox metabolism.

## Results

### The reaction stoichiometry and kinetics were studied in two reaction setups

**Figure 1:**
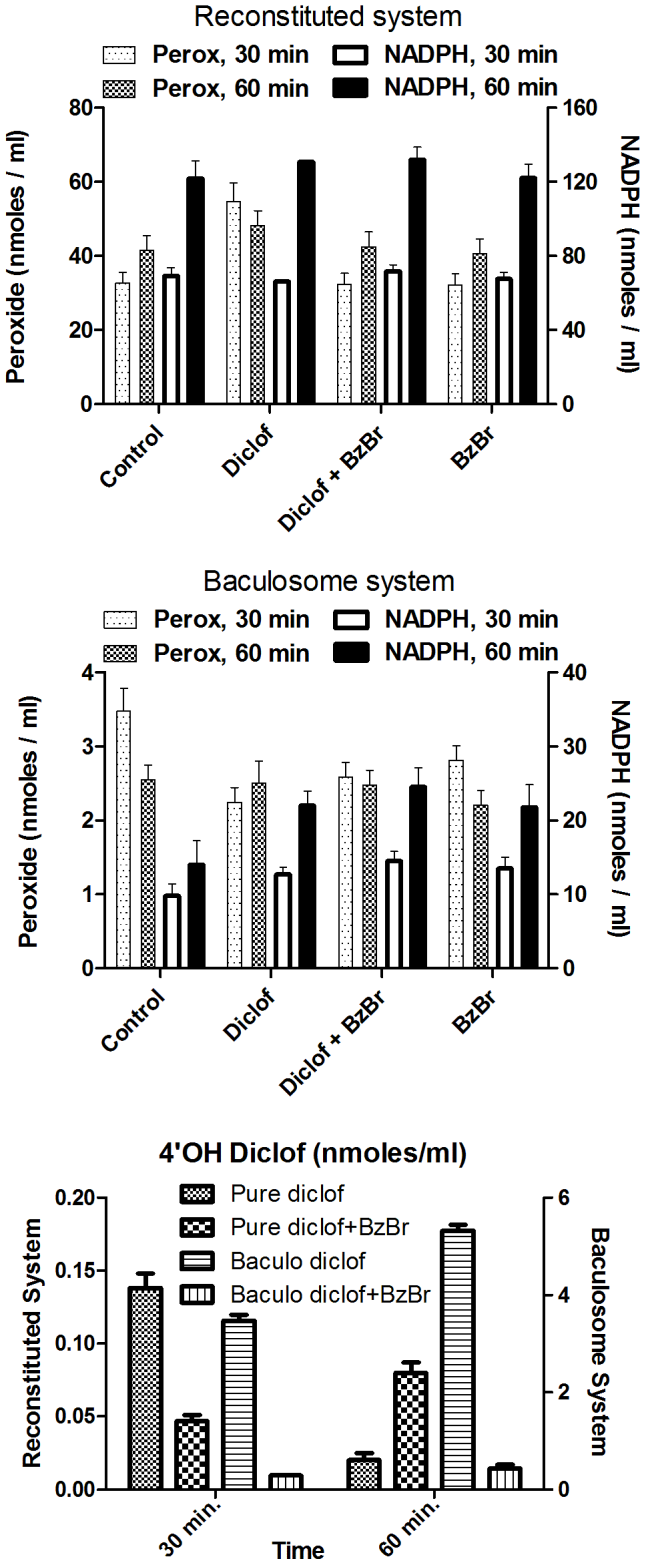
Comparing the effect of BzBr on CYP2C9 reaction dynamics in pure reconstituted and complex baculosome setups. In both setups, CYP2C9 was at 10& BzBr were at 200 µM, 200 µM and 10 µM respectively.

The highly efficient, but complex baculosomes and a reconstituted mixture of pure recombinant proteins. Figure 1 shows the overall hydroxylated diclofenac product formation in both setups in the bottom panel. The results for NADPH consumption and peroxide production in these two setups are shown in the top two panels. The reconstituted system consumed more NADPH and produced more peroxide in comparison to the baculosome setups. In contrast, the baculosome system showed a significant change in NADPH consumption rate upon the inclusion of substrates. It produced more hydroxylated product with lesser NADPH consumption and peroxide formation than the reconstituted setup. Presence of BzBr alone or its presence in conjunction with the substrate did not stop the consumption of NADPH or production of peroxide in both setups. At initial time-frame of 30 minutes, inclusion of BzBr (test reaction) gave only ∼1/12 of the control's activity in baculosomes, in comparison to the lower inhibition in reconstituted system, where the test reaction showed ∼1/3 of the control's activity. Inclusion of BzBr in reaction system significantly lowered secondary oxidation of the product in both setups, more prominently in reconstituted setup.

**Figure 2:**
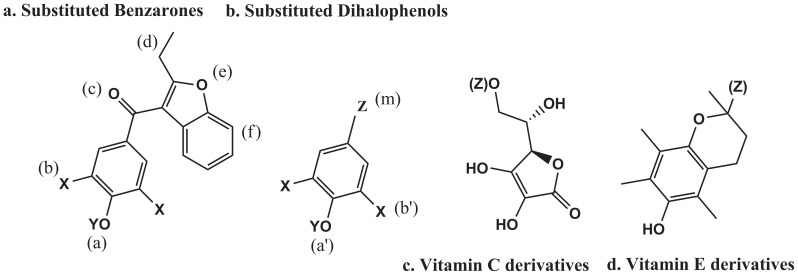
Molecular schema of various substituted benzarones, dihalophenols and vitamins studied in the current work. (For details of substitutions, please refer **Table 1**.)

**Figure 3:**
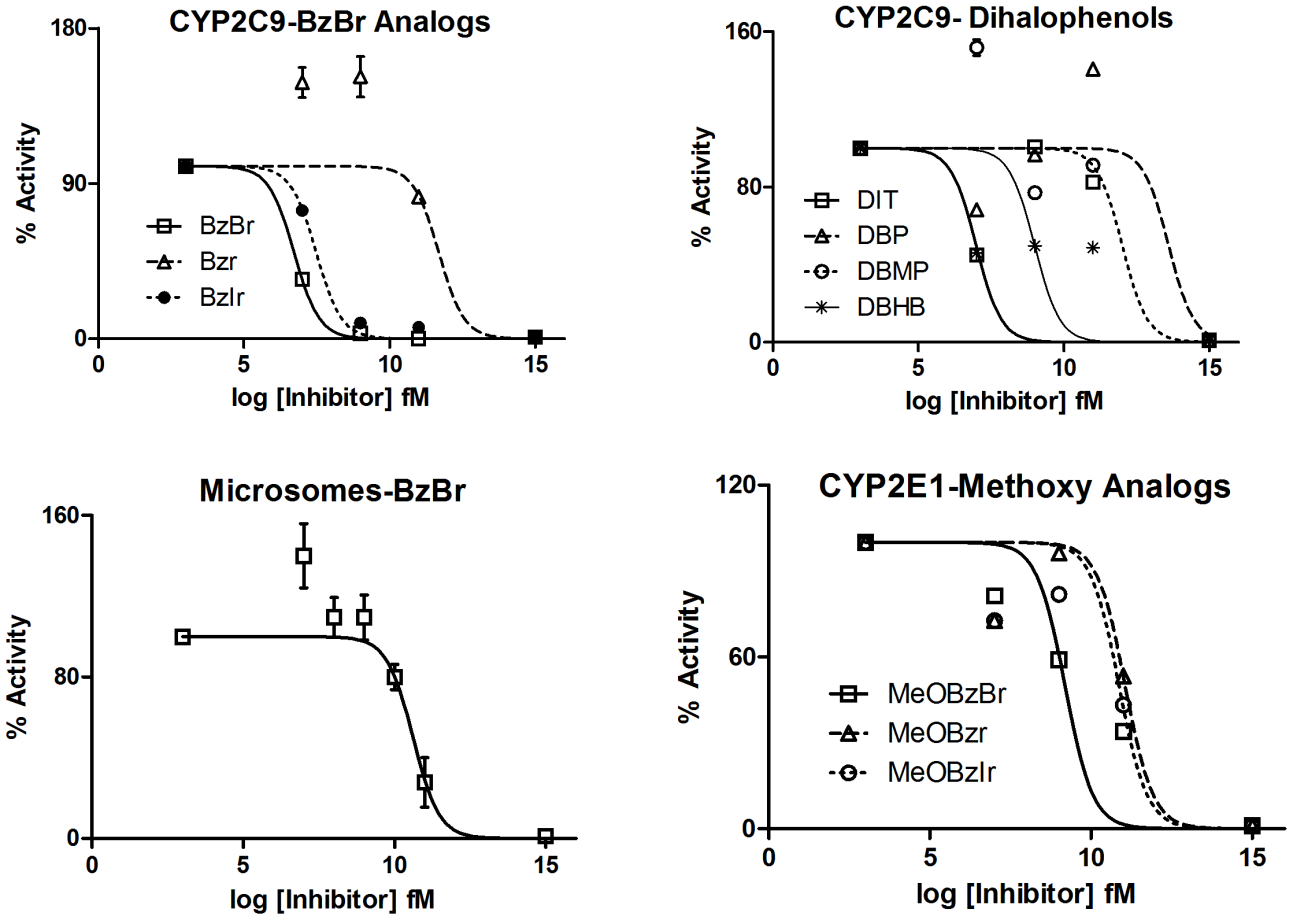
Dose-response plots for select BzBr analogues in various reaction setups of CYPs. The curves plotted are non-linear regression fits for the experimental points (with the top and bottom points weighted to facilitate curve fitting) for the equation: Y = Bottom+(Top−Bottom)/[1+10∧{(X−LogIC_50_)}].

Dose-response graphs for data points did not afford classical inhibition profiles in either CYP2C9-diclofenac (Diclof) or CYP2E1-para-nitrophenol (pNP) reactions, for both baculosome or mixed microsome systems (for most of the BzBr analogues, Figure 2 & items 1 through 10 of Table 1). Figure 3 shows some salient data profiles that could give a visual indication towards the same. A unidirectional correlation of increasing inhibitory effect was not seen upon increasing inhibitor concentrations. This observation challenges the very basis of an active site-based inhibition mechanism. Though all ten molecules were studied for determination of inhibition constants, a globally comprehensive K_i_ value determination (*i.e.*, all concentrations of inhibitors tested should show only inhibition in a given setup) was derivable only for a few reaction setups. The values so obtained are given in Table 2 [7]. Many reaction setups did not show convergence of non-linear regression plots (in spite of provision of weighted points) and/or a negative slope in linear regression analysis could not be obtained. Further, the two methodologies adopted did not give values that agreed with each other in absolute terms. For example, in CYP2C9 baculosomes, while DBHB gave a sub-nanomolar K_i_ via non-linear regression, the linear regression analysis gave a supra-millimolar K_i_ with the same experimental data.

**Table 1:** Details of substititions, characteristics and *in silico* binding analyses of various substituted benzarones, dihalophenols & vitamins with CYP2C9 (1R9O).

Inhibitor	X	Y	Z	pK_a_	log P, log D	Lowest ΔG, kcal/mol	Major Interactions	Proximal atom to Fe (Å)
***1. Bzr***	H	H	(2-ethyl-3-benzofuranyl) methanone	7.80	4.01	−8.7 (−8.8)	Arg 108, Asn 204	8.3, b
***2. BzBr***	Br	H	*Ditto*	5.11	5.55, 3.88	−9.5 (−9.5)	Arg 108, Asn 204	5.9, b
***3. BzIr***	I	H	*Ditto*	5.61	5.87, 4.71	−9.2 (−9.4)	Arg 108, Asn 204	5.7, b
***4. MeOBzr***	H	Me	*Ditto*	-	4.16	−8.7	Arg 108, Asn 204	7.8, a
***5. MeOBzBr***	Br	Me	*Ditto*	-	5.69	−9.2	Arg 108, Asn 204	6.0, b
***6. MeOBzIr***	I	Me	*Ditto*	-	6.01	−9.4	Arg 108, Asn 204	5.8, b
***7. DBP***	Br	H	H	8.43	3.21	−5.1 (−5.0)	Val 113, Leu 362	4.8, m
***8. DBMP***	Br	H	OCH_3_	8.69	3.05	−5.3 (−5.3)	Ser 209, Asn 474	8.0, a'
***9. DBHB***	Br	H	COOH	4.10, 6.96	2.86, −1.08	−5.7 (−5.7)	Ser 209, Asn 474	8.5, a'
***10. DIT***	I	H	CH_2_CHNH_2_-COOH	7.25	0.37, 0.89	−6.4 (−6.2)	Arg 108, Asp 293	4.5, b'
***Vit. C***	-	-	H	4.36, 11.20	−1.91, −4.92	−5.3	Arg 108, Asn 204	7.7, z (O)
***LAAP***	-	-	COC_15_H_31_	4.45, 11.17	5.01, 2.1	−7.2	Arg 108, Asp 293, Asn 204	5.3, z (βC)
***Vit. E***	-	-	C_16_H_33_	10.80	10.51	−9.9	Leu 362, Val 113	4.9, (OH)
***Trolox***	-	-	COOH	3.77, 12.10	3.66, 0.67	−7.7	Leu 362, Val 113	6.8, (OH)

**Table 2:** Survey of global values of K_i_ (in µM).

System	BzBr	BzIr	MeO-Bzr	MeO-BzIr	DBHB	DIT
***CYP2C9+Diclof***	0.0005 (0.90)	0.0028 (0.99)	-	-	0.0008 (−1.37)	-
	0.018 (0.99)	30.9 (0.94)	-	-	1291 (0.76)	-
***Microsomes+Diclof***	-	-	0.0007 (0.08)	0.0424 (0.25)	0.0003 (−0.59)	11.4 (−1.87)
	-	-	0.242 (0.88)	857 (0.88)	975 (0.75)	6457 (0.65)

Data obtained by non-linear (top sub-row) and linear (bottom sub-row) regression analysis (taking K_M_ to be 10 µM [7], with weighted point at pM inhibitor giving 99.9% activity), along with the R^2^ values (in brackets). A non-entry in the table means that at certain concentrations of the inhibitor molecule (in a given setup), the product formation was enhanced.

### K_i_ value calculated was significantly lower with greater order of dilution of the inhibitor, when the values were calculated with Cheng-Prusoff and Chance approaches

Table 3 shows a detailed examination of K_i_ values determined at each point, for the molecules that showed significant inhibition in many setups. At 100 µM concentration of MeOBzBr, a K_i_ value 3.7 µM was obtained in microsome setups, which is close to the value of BzBr under identical conditions. In DBP-CYP2C9 baculosome system (and also in mixed microsomes), employment of 100 µM concentration of DBP failed to give any inhibition whereas 1 µM and 10 nM levels of DBP afforded micromolar and nanomolar K_i_ values, respectively. These findings are incompatible with an active-site model of inhibition.

**Table 3:** Localized values of K_i_ (in µM) for dihalogenated phenolics calculated by Cheng-Prusoff (left sub-column) and Chance (right sub-column) equations.

System	[I] (µM)	BzBr		BzIr		DBP		DBMP		DBHB		DIT	
***CYP2C9+Diclof***	**100**	0.0009	0.01	0.65	190	-	-	95.41	342531	8.682	9332	42.62	101788
	**1**	0.0029	0.104	0.0091	0.995	2.61	99540	0.3059	1140	0.0899	97.72	-	-
	**0.01**	0.0005	0.0014	0.0026	0.243	0.002	0.101	-	-	0.0008	0.0064	0.0007	0.0055

A non-entry in the table means that the the product formation was enhanced in that particular reaction setup. (Conditions are similar to the ones in Table 2.)

### BzBr was found to inhibit the non-specific cross reactivity of various CYPs with diclofenac (an efficient substrate for CYP2C9) and pNP (an efficient substrate for CYP2E1)

Benzothiadiazole, a small planar molecule that differed structurally from BzBr and would be expected to bind to the heme-center and react competitively, was taken as a non-specific and generic inhibitor for comparison. The results of relative activities are summarized in Table 4. Submicromolar K_i_ values were derived from calculations upon incubation of enzyme reaction mixture with equivalent substrate-Bzbr concentrations, for the various CYPs tested. Further, it was seen that for pNP reactions mediated by CYP2E1 baculosomes, inhibition by molecules (taken at 10 nM concentration) like BzBr, BzIr, DBP, DBMP and DBHB gave K_i_ values in low nM ranges (calculated by the Cheng- Prusoff method).

**Table 4:** Non-specificity of inhibitions by BzBr.

Substrate	CYP2C9			CYP3A4			CYP2D6			CYP2E1		
***Diclof***	100	0.4	90.1	16	∼0.2	17	0.3	0.001	0.3	nd	nd	nd
***pNP***	nd	nd	nd	20	∼3	∼15	17	∼5	∼15	100	60	15

The relative percentage of hydroxylated product formed is shown in comparison to the positive controls. In each CYP column, first sub-column is without any inhibitor, second and third sub-columns are with an equivalent amount of BzBr and benzothiadiazole respectively. Concentrations of baculosomes- 20 nM CYP, 250 µM NADPH and 80 µM Diclof or pNP. Sampling for hydroxylated product analysis was at 30 minutes. [*nd: product formed was too low to be detected under the reaction assay conditions].

### *In silico* exploration of BzBr analogues with CYP2C9 active site does not provide support for a binding-based inhibition

**Figure 4:**
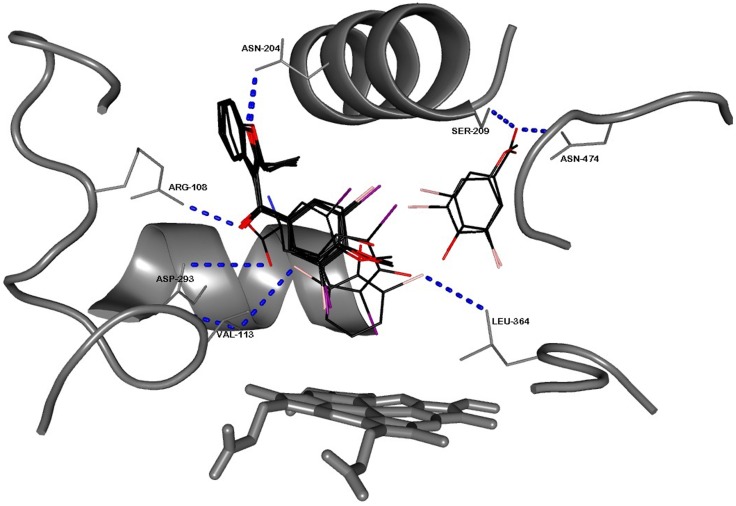
Snapshots of the ten benzarone/dihalophenolic analogues docked in the active site of CYP2C9 (1R9O).

The binding scenarios of two soluble CYP enzyme-substrate sets and membrane protein CYP2C9 with warfarin/flurbiprofen sets (Material A(1) in File S1: Supporting Information) showed very similar docking as the crystal structures of the same protein-ligand sets, thereby validating the *in silico* investigations. Known heme-enzyme+specific substrate combinations like P450cam+camphor and P450BM3+palmitic acid gave binding energies comparable to diverse molecules studied for liver microsomal CYP2C9 (Material A(2) in File S1: Supporting Information). Binding energies for substrates/inhibitors were typically found to depend more on the extent of hydrophobicity and the size of the small molecule and were practically independent of any particular recognition mechanism within the active site. It is sometimes proposed that primary binding of an inhibitor molecule could prevent the substrate molecule from binding secondarily. Such scenarios were also explored. The bound inhibitory ligand could not prevent another substrate molecule from binding, as the catalytic site was spatially large enough to accommodate both molecules. Arg 108 did not play a significant role in the binding of dapsone (a ligand known to enhance CYP2C9's activity [8]) and some of the smaller dihalophenolics. Also, the binding of activators and inhibitors did not correlate to logically predictable alterations in binding energy or proximity to the heme center for the subsequent binding of substrates. Diverse substrates of CYP2C9 gave comparable binding energies to those for the BzBr analogues tested. Binding of anionic ligands did not give any change in binding energy or any altered orientation in the 1R9O active site, either for the original substrates or for competitive inhibitory ligands (Table 1, values given in parentheses). Inhibition was also seen for much smaller molecules that bound at loci other than where BzBr bound. The “relatively inefficient inhibitors” in meth(ox)yl derivatives of Bzr, BzBr and BzIr bound in practically identical fashion to their hydroxylated parent molecules (Figure 4), giving similar binding energies and proximity to the heme center (Table 1).

### Known amphipathic radical scavengers critically inhibited CYP2C9 reactions

**Figure 5:**
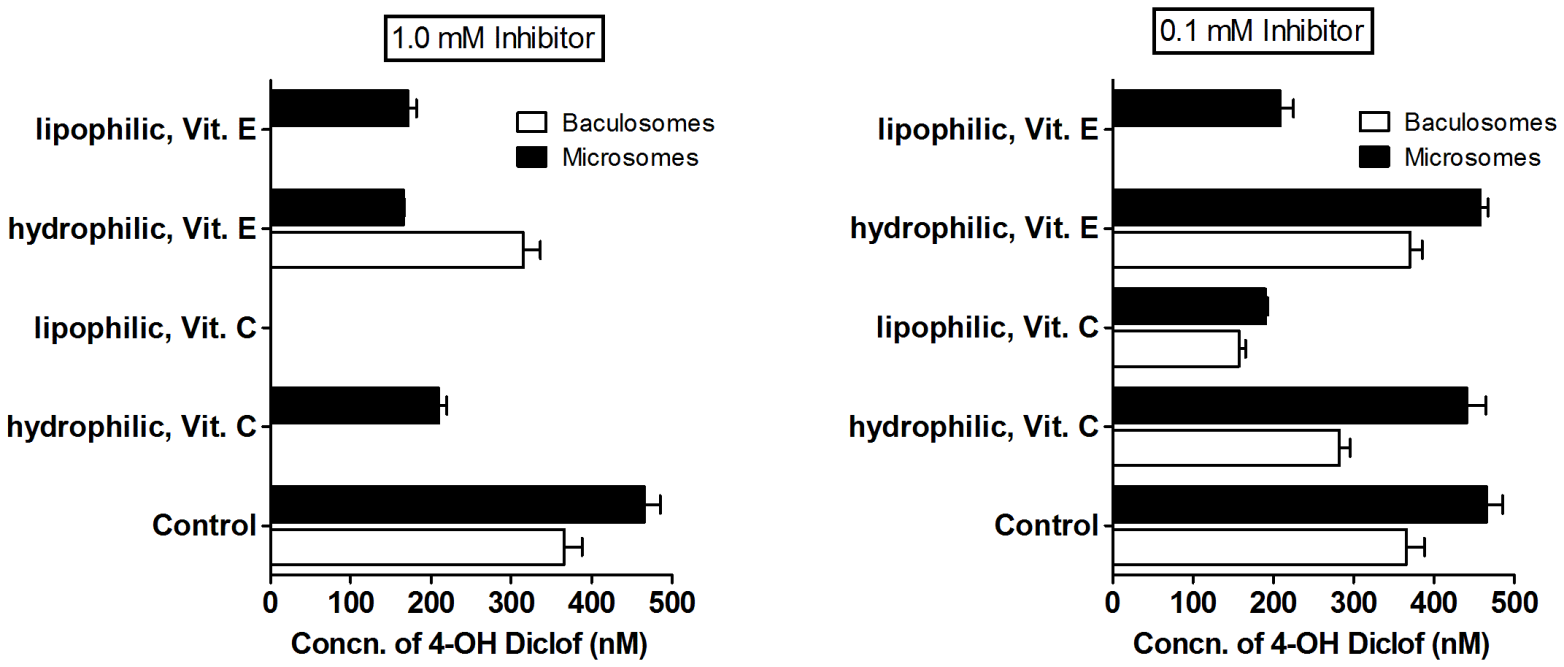
Vitamins-mediated inhibition of CYP2C9 baculosomes and microsome reactions for hydroxylation of diclofenac. The reactions were carried out in 100 µM diclofenac, 200 µM NADPH, rat liver microsomes with 0.5 µM of CYPs or 10 nM of CYP 2C9 baculosome preparation, taken along with the appropriate concentration of the redox molecule. The plots show 4′OH diclofenac in milieu. after 10 minutes reaction time.

Polar vitamin C, its amphipathic fatty acyl derivative (L-ascorbic acid palmitate or LAAP), hydrophobic vitamin E and its truncated soluble derivative (Trolox) were employed for tracing the interfacial distribution effects of radical scavengers on the CYP2C9 reaction. As seen from Figure 5, mM concentrations of vitamins C & E (and their derivatives) could bring about significant inhibition of CYP2C9 activities in both the systems. An order of magnitude lower concentration of the same molecules (at equivalent concentration to the original substrates) gave effective inhibition (>50% of the control) only in the lipophilic derivatives of the vitamins. At higher concentrations of the inhibitors, since the relatively hydrophilic molecule of vitamin C is a more effective inhibitor than the soluble derivative of vitamin E, it is highly improbable that the overall inhibition is owing to an active site binding effect. Vitamin E, though structurally very different from benzbromarone, also gave ∼nM K_i_ in the baculosome reaction setup.

## Discussion

Generally, a stronger binding affinity results from multiple interactions between proteins' amino acid residues and different moieties within the small molecule (ligand), as exemplified by *in silico* studies of the streptavidin-biotin binding where at least six amino acid residues (Asn 23, Ser 27, Tyr 43, Ser 45, Ser 88, Asp 128) of the protein interact with the small molecule. The active site model of inhibition for CYP with BzBr analogues is incapable of explaining how and why the presence of- (i) a dissociable proton or (ii) two halogen atoms or (iii) some small substitutions etc. would alter the functional inhibition in such a drastic fashion. More importantly, it cannot explain how the very same inhibiting molecule changes to an activator at certain concentrations (for example- MeOBzBr and DBMP, Tables 2 & 3). The fact that the surface-active molecule of BzBr is a better inhibitor in the baculosome system compared to reconstituted system containing pure components, points to the role of the microenvironment of the reaction system in determining the reaction outcome. Considering the data of Table 4, Occam's razor argues for a more generic and active site-disconnected phenomenon. It is very difficult to envisage that BzBr has high affinities for diverse active sites of various CYP, and competitively “outbinds” different substrates with varying topography, all the while giving very low K_i_ values. With *in silico* studies too, positive support for the binding model was lacking. Conclusive argument against the active-site model is derived from the studies incorporating structurally diverse redox-active vitamins and their derivatives in two different reaction setups. Since hydrophobic derivatives of the vitamins gave effective inhibition in both microsome and baculosome setups (Figure 5), the basic determining process must occur at the phospholipid interface.

**Figure 6:**
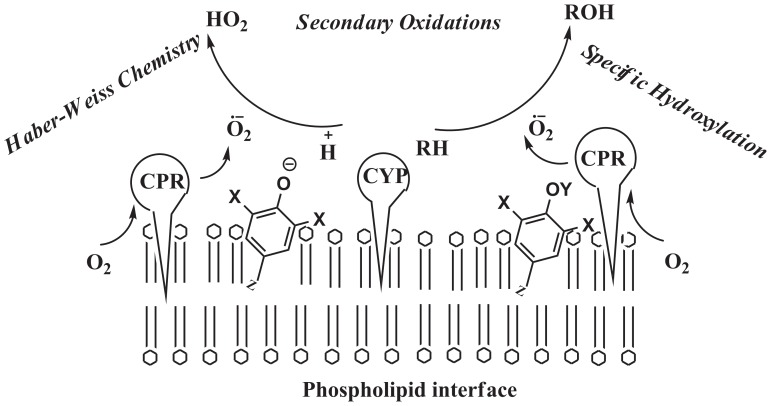
A scheme proposed to explain the probable interactions and networks of various reaction components in the liver microsomal CYP system. (Details given in text.)

Therefore, obtaining a sub-nM K_i_ with low concentrations of inhibitors (at supra-nM levels of enzymes) was found to depend more on the presence of the positioning of a radical scavenger on the phospholipid membrane, where CYP and its reductase (CPR) are ‘housed’. A non-specific interfacial catalytic mechanism, as depicted in Figure 6, could help explain the effects observed. The enzyme cytochrome P450 reductase (CPR) produces DROS (diffusible reduced oxygen species, as exemplified by superoxide shown) from oxygen at the lipid interface [9], [10]. These DROS, when localized around the phospholipid interface, could generally be utilized by CYP to hydroxylate the substrate. Further, the DROS species are in equilibrium with several other molecular species (in both phases) and involved in Haber-Weiss chemistry. It is also known that a DROS such as superoxide is not considered to be a highly active species but its protonated form is [11]–[13]. BzBr could serve as an interim catalyst, modulating the concentrations of DROS, along the lines mentioned in our recent works [14]–[16]. The two halogen moieties aid in effective interfacial positioning. The stability of the lipid soluble radicals coupled with the availability of a water-soluble proton makes the process a classical interfacial reaction, which BzBr (and such molecules) can bring together. For the same concentrations of substrate and enzyme, better inhibition efficiencies are seen in select systems with low amounts of BzBr analogues because of the stability of radical intermediates at lower concentrations. In the baculosome reaction setups with relatively higher CPR∶CYP ratios, BzBr's utilization of DROS by CPR at the lipid interface would significantly affect the well-positioned CYP-CPR redox relay machinery. In contrast to the constructive process of substrate hydroxylation in baculosomes, DROS production and the associated cross reactivity within the microenvironment is greater in the microsomes setup. In these systems, poor distribution and positioning of enzymes would not give high impact of BzBr, because of the presence of relatively higher amounts of CYPs (which could also prolong the lifetime of DROS). BzBr has the ability to alter secondary oxidation profiles of diclofenac (Figure 1) and this conclusively shows that BzBr is involved in DROS dynamics. The partitioning of the redox active molecules into the aqueous phase could also catalyze the reverse process of generating the DROS or stabilizing the same, thereby accounting for the enhancement effect. In the case of methoxy derivatives, complications can arise owing to the enzymatic or non-enzymatic demethylation of the original molecule, thereby generating small amounts of hydroxylated catalytic species from the substrate. So, we get a mixture of effects therefrom.

Since the reaction is governed by lifetimes, concentrations, pertinent equilibriums and reactivity of a wide variety of intermediates generated in milieu, it is now possible to understand the apparently chaotic modality of overall outcome. In our recent works, we had demonstrated that small concentrations of various redox active molecules could modulate one-electron reactions mediated by heme proteins [14]–[16] and stressed on their relevance in physiological realms. It is well-known that superoxide dismutase and catalase are included in CYP reaction mixtures. Small amounts of these soluble enzymes are (quite akin to the soluble radical scavengers!) inefficient in curbing the 2-dimensionally constrained interfacial reactions. They end up lowering secondary oxidations of substrate in aqueous milieu, thereby enhancing product yield. Their inability to inhibit CYP reactions is not an anti-thesis to the fundamental idea proposed herein. We had solved substrate inhibitions in several heme enzyme systems (including CYPs) [10] and the inhibitory role of CPR's truncated N-term in CYP mediated catalysis, attributing it to an interfacial DROS modulating effect [17]. This manuscript consolidates our pursuits and provides support for the obligatory role of DROS in CYP functioning. The fundamental ideas derived herein would explain the low distribution densities of CPR, the promiscuity of this single CPR serving as a partner to all CYPs, substrate diversities of CYPs and several other problematic aspects of the currently prevailing hypothesis in CYP literature (which seeks that the first step of oxygen activation occurs at the heme-center).

## Materials and Methods

### Enzyme preparations

CYP2C9 baculosomes were obtained from Life Technologies (and Panvera), Invitrogen. For pure CYP & CPR reactions, the protocols for protein preparations are described elsewhere [10]. Freshly excised rat liver was used for microsomal preparations [18] and immediately transferred to a pre-cooled homogenizing buffer. Animal procedures were approved by the Institutional Animal Ethics Committee (IAEC, VIT University), which was ratified by the Government of India vide order no. 1333/C/10/CPCSEA. Liver chunks were homogenized and then spun down at 12,000 rpm for 30 minutes at 4°C and the supernatant was collected in fresh round bottom tubes. This supernatant was run in an ultracentrifuge at 80,000 g for 90 minutes at 4°C. The pellet was then washed with phosphate buffer and finally stored in suspension buffer containing 20% glycerol. Aliquots of 1 ml were stored at −40°C until use. Protein preparations were rapidly thawed by suspending the vial in 37°C water bath just before use and then immediately transferred on ice.

### Materials

The small dihalogenated phenolics used in current study were purchased from Sigma-Aldrich. Methoxylated forms of benzarone, benzbromarone and benziodarone were synthesized in lab from the corresponding pure hydroxylated analogue molecules, which were gifted by Tonira Pharma Ltd. (India).

### Organic synthesis and characterization

Reactions were performed under an inert atmosphere of nitrogen with magnetic stirring. Syringe was used to transfer air-sensitive solvents and reagents. Acetone was distilled from potassium carbonate and stored over 4 Å molecular sieves prior to use. Analytical thin layer chromatography (TLC) was performed on MERCK precoated silica gel 60 F_254_TLC plates. Eluting solvents are reported as volume percents. Compounds were visualized using UV light and iodine stain. Flash column chromatography was performed using silica gel (200–400 mesh) from Acme chemicals. All NMR spectra (details given in Materials B in File S1: Supporting Information) were recorded on Bruker 400 spectrometer using CDCl_3_ as solvent. The NMR spectra were referenced using residual solvent peaks as the standard. Chemical shifts are denoted in parts per million (δ) and coupling constants (*J*) are reported in Hertz (Hz). The spin multiplicities are reported as singlet (s), doublet (d), triplet (t), quartet (q) and multiplet (m). High resolution mass spectra (HRMS) were recorded on MICRO-Q-TOF mass spectrometer using the ESI technique. FT-IR spectra were recorded on a JASCO FT/IR-4100 spectrometer. All IR spectra were recorded as a thin film of compounds in chloroform. IR spectra peaks are reported in wavenumbers (cm^−1^) as strong (s), medium (m), weak (w), and broad (br).

### General procedure for meth(ox)ylation of benzarone derivates

A solution of the benzarone derivative (1 equiv) in acetone (0.25M) was treated with potassium carbonate (1.1 equiv) and methyl iodide (6 equiv). The reaction mixture allowed to stir at 45°C until starting material was consumed, following which acetone was removed in vacuo. The resulting suspension was dissolved in ethyl acetate (30 mL) and washed with water (2×20 mL). The organic layer was separated, dried over anhydrous sodium sulfate, filtered and concentrated in vacuo. Purification by flash chromatography afforded the methylated produce in 99% yield.

### Enzyme reaction and product analyses

The reactions were carried out in 1 ml vial at 27±1°C in 100 mM potassium phosphate buffer at pH 7.4±0.1. A stopper mixture of 94% acetonitrile and 6% acetic acid was used to cease the reaction. Total reaction volume was 250 µl and the stopper added was 100 µl to each reaction mixture. 4′-OH diclofenac formation was observed at various time points and quantified with HPLC with detection at 278 nm [10]. CYP3A4 and CYP2D6 did not give the same reaction product profiles as CYP2C9 does with diclofenac. However, the area given under the obtained products' chromatogram peaks were calculated with similar terms. In some reactions, another method was used. Kromacil®100-5C8 reversed phase column with 5 µm particle size packing and with an internal diameter of 4.6 mm and a length of 250 mm was employed for routine HPLC. An isocratic mobile phase constituting of 0.1% trifluoroacetic acid in water, methanol and acetonitrile in the ratio 2∶2∶1 was used at 1 ml/min for elution, giving a retention time of ∼13 min for 4′-OH diclofenac and ∼30 min for diclofenac. CYP2E1 activity was assayed by HPLC methods and the data was seen to correspond well with the spectrophotometric assay [19], [20] and both these methodologies were used for the determination of pNP reaction products. For the determination of K_i_ values, in reactions with microsomes, diclofenac and NADPH concentrations were 200 µM and 400 µM respectively and total CYP concentration was 0.5 µM. Whereas in reaction with baculosomes, concentration of CYP was 10 nM, diclofenac and NADPH were at 100 µM and 200 µM respectively. The inhibitory small molecules were employed under varying orders of magnitude. The reactants were added 25 µl each from a 10X respective concentrate and the remaining volume was made up to 250 µl with double distilled deionized water. The values reported are from duplicates or triplicates, along with the standard deviations. GraphPad Prism 5.02 was employed for data analysis and graph generation.

### Calculation of K_i_

Traditionally, K_i_ is defined (for competitive binding) as the concentration of inhibitor required to alter the enzyme-substrate interaction so that the apparent K_M_ (which is the concentration of substrate at which reaction rate is half the maximal rate achievable) doubles that of original K_M_ value obtained in the absence of inhibitor. In many cases, K_M_ can also be a crude index of enzyme-substrate binding affinity. The value of K_i_ is usually calculated from experimentally determined IC_50_ values (which is defined as the concentration of inhibitor that results in 50% reduction in the rate of enzyme catalyzed reaction). IC_50_ is derived by non-linear regression analysis from a log plot of inhibitor concentration versus percentage activity. The IC_50_ value thus obtained is used to determine K_i_ using Cheng-Prusoff equation [21]-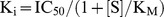
where [S] is the substrate concentration employed. A linear regression method with a double logarithmic plot is also available for the calculation of K_i_, as originally developed by Britton Chance [22]. This equation, which does not use K_M_ value, is-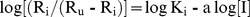
where R_i_ and R_u_ are inhibited and uninhibited rates obtained with the inhibitor concentration [I].

### *In silico* methods

Crystal structures for proteins were obtained from RCSB Protein Data Bank and that of ligand from PubChem if available. Otherwise, the ligands were designed in ChemBioDraw Ultra 12.0 and 3-D structure generated using online tool CORINA [23]. Ligands were then converted to appropriate file types using Open Babel 2.3.2 [24] and were energy minimized in Chimera 1.7 [25]. Protein and ligand were prepared for docking using AutoDock Tools (MGL Tools 1.5.4) [26], [27]. AutoDock Vina 1.1.1 was used to explore the binding sites of ligands in the protein [28]. Keeping the grid size at 23 for all, different regions of protein were screened to identify the best binding site based on the lowest binding energy. The output was visualized and exported for image with PyMol 1.3 [29].

## Supporting Information

File S1 Figure S1: CYP2C9 (1R9O) – Flurbiprofen. Docked flurbiprofen aligned with the crystal structure of flurbiprofen bound CYP2C9. Figure S2: CYP2C9 (1OG5) – Warfarin. Docked S-warfarin (green) aligned with the crystal structure of flurbiprofen bound CYP2C9. Figure S3: P450cam – Camphor. Aligned camphor bound P450cam crystal structure with the docked camphor. Figure S4: P450BM3- Palmitic acid. Docked palmitic acid represented in alignment with palmitate bound crystal structure of P450BM3. Figure S5: NMR (^1^H) spectra for MeOBzr. Figure S6: NMR (^13^C) spectra for MeOBzr. Figure S7: NMR (^1^H) spectra for MeOBzBr. Figure S8: NMR (^13^C) spectra for MeOBzBr. Figure S9: NMR (^1^H) spectra for MeOBzIr. Figure S10: NMR (^13^C) spectra for MeOBzIr. Table S1: In silico docking analyses of some CYPs (with known crystal structures) for diverse substrates, activators and inhibitors.
(DOC)

## References

[1] Wester MR, Yano JK, Schoch GA, Yang C, Griffin KJ, et al (2004) The structure of human cytochrome P450 2C9 complexed with flurbiprofen at 2.0-Å resolution. J Biol Chem 279: 35630–35637. 15181000 10.1074/jbc.M405427200

[2] Dickmann LJ, Locuson CW, Jones JP, Rettie AE (2004) Differential roles of Arg97, Asp293, and Arg108 in enzyme stability and substrate specificity of CYP2C9. Mol Pharmacol 65: 842–850. 15044613 10.1124/mol.65.4.842

[3] Williams PA, Cosme J, Ward A, Angove HC, Vinkovi, et al (2003) Crystal structure of human cytochrome P450 2C9 with bound warfarin. Nature 424: 464–468. 12861225 10.1038/nature01862

[4] Locuson CW, Wahlstrom JL, Rock DA, Rock DA, Jones JP (2003) A new class of CYP2C9 inhibitors: probing 2C9 specificity with high-affinity benzbromarone derivatives. Drug Metab Dispos 31: 967–971. 12814975 10.1124/dmd.31.7.967

[5] Locuson CW, Rock DA, Jones JP (2004) Quantitative binding models for CYP2C9 based on benzbromarone analogues. Biochemistry 43: 6948–6958. 15170332 10.1021/bi049651o

[6] Peng C-C, Rushmore T, Crouch GJ, Jones JP (2008) Modeling and synthesis of novel tight-binding inhibitors of cytochrome P450 2C9. Bioorg Med Chem 16: 4064–4074. 18255300 10.1016/j.bmc.2008.01.021

[7] Bort R, Macé K, Boobis A, Gómez-Lechón Ma-J, Pfeifer A, et al (1999) Hepatic metabolism of diclofenac: role of human *CYP* in the minor oxidative pathways. Biochem Pharmacol 58: 787–796. 10449188 10.1016/s0006-2952(99)00167-7

[8] Hutzler JM, Kolwankar D, Hummel MA, Tracy TS (2002) Activation of CYP2C9-mediated metabolism by a series of dapsone analogs: kinetics and structural requirements. Drug Metab Dispos 30: 1194–1200. 12386124 10.1124/dmd.30.11.1194

[9] Manoj KM, Gade SK, Mathew L (2010) Cytochrome P450 Reductase: A Harbinger of Diffusible Reduced Oxygen Species. PLoS One 5: e13272. 20967245 10.1371/journal.pone.0013272 PMC2954143

[10] Manoj KM, Baburaj A, Ephraim B, Pappachan F, Maviliparambathu PP, et al (2010) Explaining the atypical reaction profiles of heme enzymes with a novel mechanistic hypothesis and kinetic treatment. PLoS One 5: e10601. 20498847 10.1371/journal.pone.0010601 PMC2871781

[11] Sawyer DT, Valentine JS (1981) How super is superoxide? Acc Chem Res 14: 393–400.

[12] De Grey AD (2002) HO_2_*: the forgotten radical. DNA Cell Biol 21: 251–257. 12042065 10.1089/104454902753759672

[13] Blanksby SJ, Bierbaum VM, Ellison GB, Kato S (2007) Superoxide does react with peroxides: direct observation of the Haber–Weiss reaction in the gas phase. Angew Chem 46: 4948–4950. 17514688 10.1002/anie.200700219

[14] Andrew D, Hager L, Manoj KM (2011) The intriguing enhancement of chloroperoxidase mediated one-electron oxidations by azide, a known active-site ligand. Biochem Biophys Res Commun 414: 646–649. 10.1016/j.bbrc.2011.10.128 22079633

[15] Gade SK, Bhattacharya S, Manoj KM (2012) Redox active molecules cytochrome *c* and vitamin C enhance heme-enzyme peroxidations by serving as non-specific agents for redox relay. Biochem Biophys Res Commun 419: 211–214. 22342667 10.1016/j.bbrc.2012.01.149

[16] Parashar A, Manoj KM (2012) Traces of certain drug molecules could enhance heme-enzyme catalytic outcomes. Biochem Biophys Res Commun 417: 1041–1045. 22214932 10.1016/j.bbrc.2011.12.090

[17] Gideon DA, Kumari R, Lynn AM, Manoj KM (2012) What is the Functional Role of N-terminal Transmembrane Helices in the Metabolism Mediated by Liver Microsomal Cytochrome P450 and its Reductase? Cell Biochem Biophys 63: 35–45. 10.1007/s12013-012-9339-0 22302675

[18] Lake BG (1987) Preparation and characterisation of microsomal fractions for studies on xenobiotic metabolism. In: Snell K, Mullock B, editors. Biochemical Toxicology: A Practical Approach. Illustrated ed: IRL Press. pp. 183–216.

[19] Reinke L, Moyer M (1985) p-Nitrophenol hydroxylation. A microsomal oxidation which is highly inducible by ethanol. Drug Metab Dispos 13: 548–552. 2865101

[20] Chang TK, Crespi CL, Waxman DJ (1998) Spectrophotometric analysis of human CYP2E1-catalyzed p-nitrophenol hydroxylation. Methods Mol Biol 107: 147–152. 14577224 10.1385/0-89603-519-0:147

[21] Yung-Chi C, Prusoff WH (1973) Relationship between the inhibition constant (*K_I_*) and the concentration of inhibitor which causes 50 per cent inhibition (*I_50_*) of an enzymatic reaction. Biochem Pharmacol 22: 3099–3108. 4202581 10.1016/0006-2952(73)90196-2

[22] Chance B (1943) The effect of cyanide on the kinetics of the enzyme-substrate compound and overall reaction of peroxidase. J Cell Comp Physiol 22: 33–41.

[23] Gasteiger J, Rudolph C, Sadowski J (1990) Automatic generation of 3D-atomic coordinates for organic molecules. Tetrahedron Comput Meth 3: 537–547.

[24] O'Boyle NM, Banck M, James CA, Morley C, Vandermeersch T, et al (2011) Open Babel: An open chemical toolbox. J Cheminform 3: 1–14. 21982300 10.1186/1758-2946-3-33 PMC3198950

[25] Pettersen EF, Goddard TD, Huang CC, Couch GS, Greenblatt DM, et al (2004) UCSF Chimera- a visualization system for exploratory research and analysis. J Comput Chem 25: 1605–1612. 15264254 10.1002/jcc.20084

[26] Morris GM, Huey R, Lindstrom W, Sanner MF, Belew RK, et al (2009) AutoDock4 and AutoDockTools4: Automated docking with selective receptor flexibility. J Comput Chem 30: 2785–2791. 19399780 10.1002/jcc.21256 PMC2760638

[27] Sanner MF (1999) Python: a programming language for software integration and development. J Mol Graph Model 17: 57–61. 10660911

[28] Trott O, Olson AJ (2010) AutoDock Vina: improving the speed and accuracy of docking with a new scoring function, efficient optimization, and multithreading. J Comput Chem 31: 455–461. 19499576 10.1002/jcc.21334 PMC3041641

[29] DeLano W (2002) The PyMOL Molecular Graphics System. DeLano Scientific; San Carlos, CA, USA.

