# Supplementary material for: The Curious Case of Benzbromarone: Insight into Super-Inhibition of Cytochrome P450
Source: PLoS One. 2014 Mar 3;9(3):e89967. doi: 10.1371/journal.pone.0089967 (PMC3940698; doi:10.1371/journal.pone.0089967)

**FILE S1: SUPPORTING INFORMATION**

**Material A(1): Close resemblance of the docked ligands and CYPs (a comparison with available crystal structures)**

**Figure S1: CYP2C9 (1R9O) - Flurbiprofen**


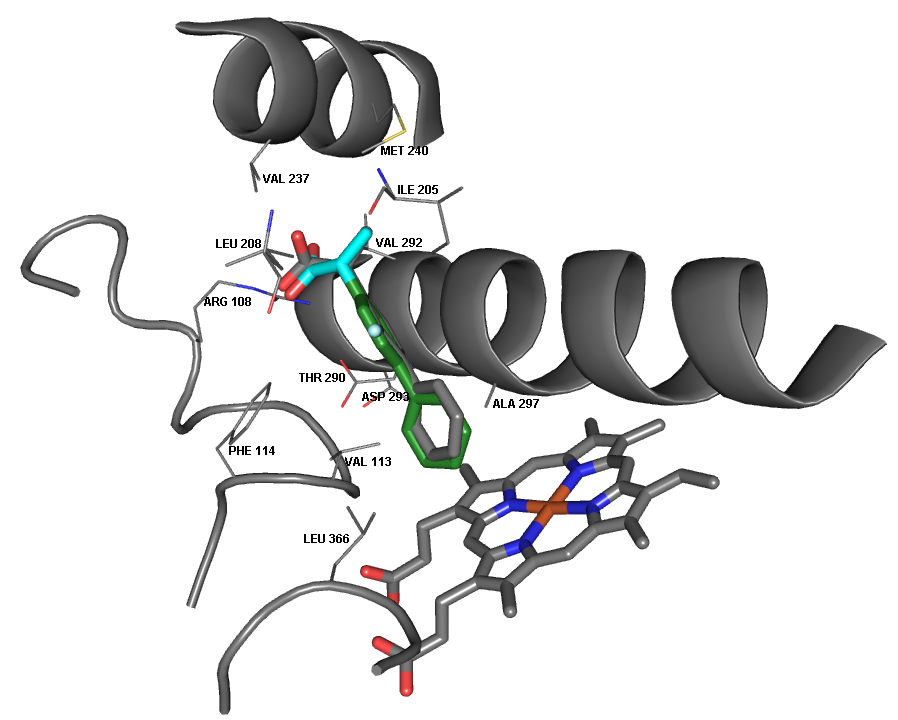


Figure S1: Docked flurbiprofen (green) aligned with the crystal structure of flurbiprofen bound CYP2C9 with the accession number 1R9O (from RCSB). The interacting amino acids have been labeled along with the heme shown at the base of ligand.

**Figure S2: CYP2C9 (1OG5) - Warfarin**


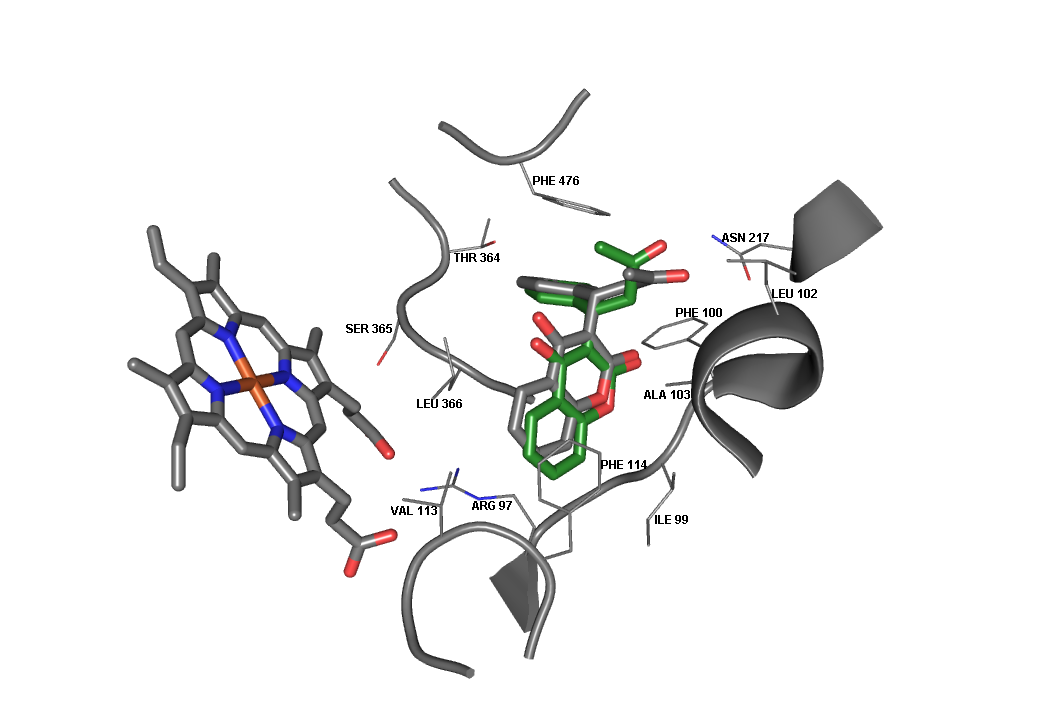


Figure S2: Docked S-warfarin (green) aligned with the crystal structure of flurbiprofen bound CYP2C9 with the accession number 1OG5 (from RCSB). The interacting amino acids have been labeled along with the heme shown at the base of ligand.

**Figure S3: P450cam - Camphor**


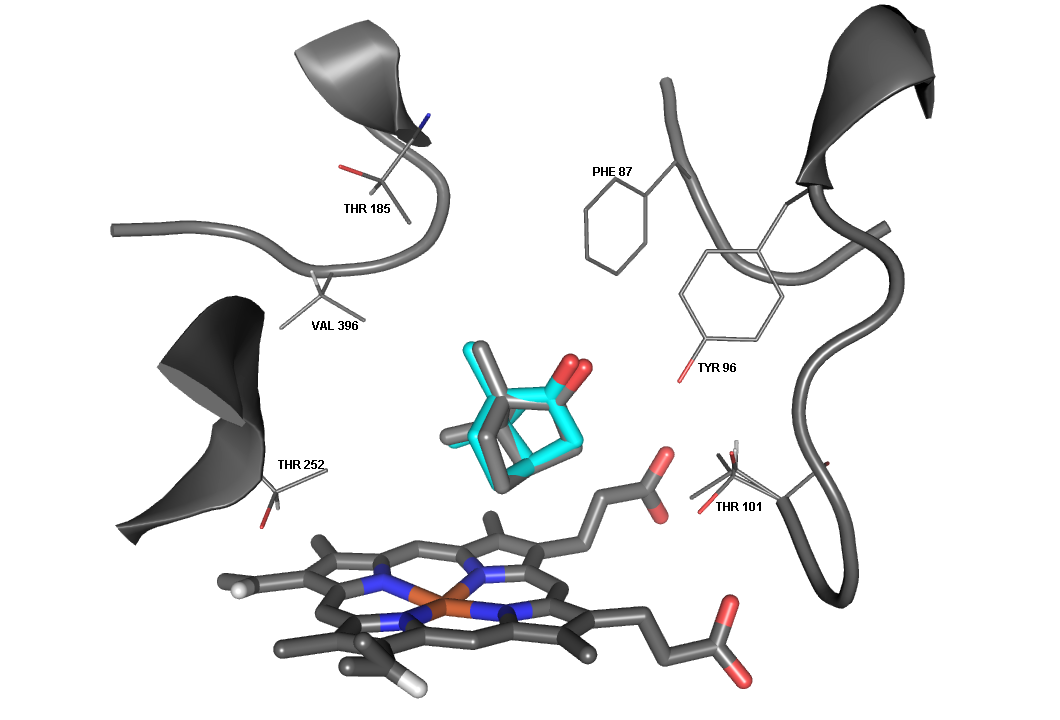


Figure S3: Aligned camphor bound P450cam crystal structure (2ZWT) with the docked camphor (shown in cyan).The active site residues involved in substrate recognition and interaction is labeled and shown in the image.

**Figure S4: P450BM3- Palmitic acid**


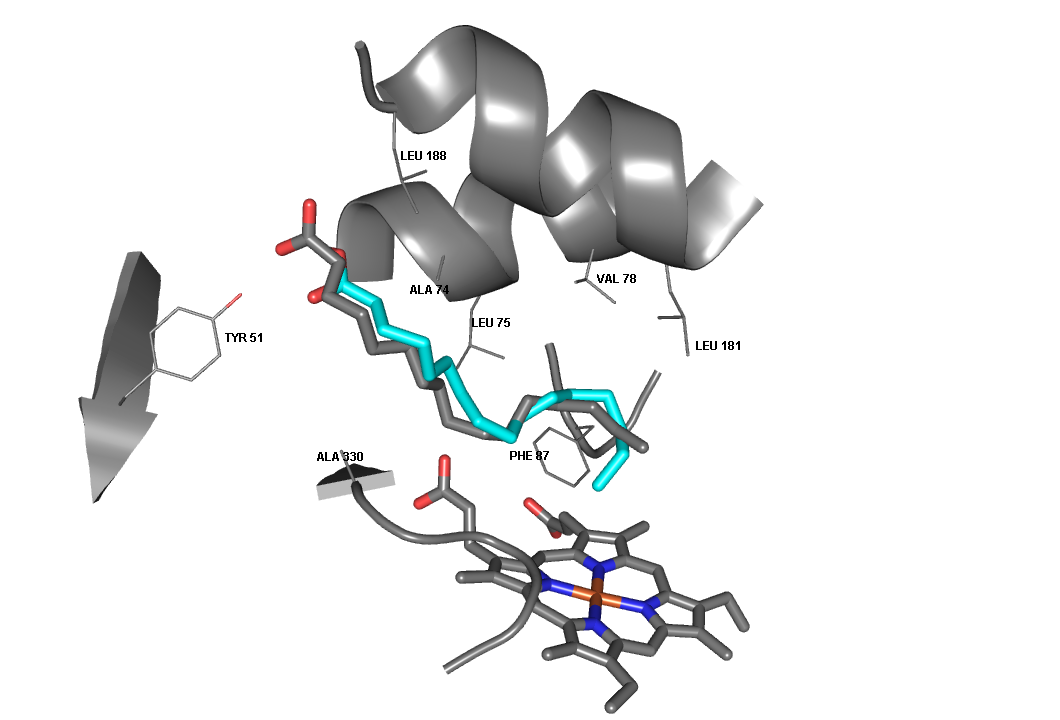


Figure S4: Docked palmitic acid (cyan) has been represented in alignment with palmitate bound crystal structure of P450BM3 (2UWH). The amino acids associated with interaction and recognition of the ligand has been labeled and shown.

**Material A(2): *In silico* docking analyses of some CYPs (with known crystal structures) for diverse substrates, activators and inhibitors (Table S1):**

Table S1:

| **Receptor Molecule-**  **PDB Name** | **Ligand** | **Lowest Free Energy (ΔG) kcal/mol** | **Major Interactions** | **Distance of Reaction Site (and proximal atom) to Fe (Å)** |
| --- | --- | --- | --- | --- |
| ***P450cam-2ZWT*** | Camphor | -8.1 | Tyr 96 | 5.1 (3.6) |
| ***P450BM3-1BVY*** | Palmitic acid | -5.5 | Tyr 51 | 5.7 (5.7) |
| ***CYP2C9-1OG5*** | S-Warfarin | -7.98 | Phe 100, Asn 217 | 11.9 (10.6) |
| ***CYP2C9-1R9O*** | Flurbiprofen | -6.23 | Arg 108 | 4.4 (4.4) |
| ***CYP2C9-1R9O*** | S-Warfarin | -6.23 | Arg 108, Gly 296 | 3.4 (3.4) |
| ***CYP2C9-1R9O*** | Ibuprofen | -6.8 | Arg 108, Asp 293 | 4.3 (4.3) |
| ***CYP2C9-1R9O*** | Amiodarone | -6.59 | Arg 108, Asn 204 | 9.5 (6.2) |
| ***CYP2C9-1R9O*** | Dapsone | -5.65 | Ile 205, Ala 297 | 2.8 (2.1) |
| ***CYP2C9-1R9O*** | Losartan | -7.58 | Arg 108; Phe 100 | 11.1 (3.8) |
| ***CYP2C9-1R9O*** | Sildenafil | -8.6 | Arg 108 | 12.1 (5.3) |
| ***CYP2C9-1R9O*** | Piroxicam | -8.8 | Arg 108 | 8.0 (4.4) |
| ***CYP2C9-1R9O*** | Naproxen | -7.4 | Arg 108; Phe 114 | 5.3 (4.7) |
| ***CYP2C9-1R9O*** | Diclofenac | -7.5 | Phe 100, Phe 114, Phe 476 | 4.7 (4.7) |
| ***Flurbiprofen bound CYP2C9-1R9O*** | Amiodarone | -2.6 | Ser 209; Thr 304; Arg 307; Asn 474 | 19.7 (10.2) |
| ***Flurbiprofen bound CYP2C9-1R9O*** | Dapsone | -5.51 | Gly 296; Phe 476 | 7.2 (6.5) |
| ***Flurbiprofen bound CYP2C9-1R9O*** | BzBr | -7.4 | Phe 100, Phe 476 | 16.8 (10.0) |
| ***Flurbiprofen bound CYP2C9-1R9O*** | DBHB | -6.2 | Ser 209, Asn 474 | 8.4 (8.4) |
| ***Amiodarone bound CYP2C9-1R9O*** | Flurbiprofen | -6.79 | His 230; Phe 100 | 15.2 (14.6) |
| ***Dapsone bound CYP2C9-1R9O*** | Flurbiprofen | -7.00 | Arg 108, Asn 204; Phe 100, Phe 114 | 17.0 (10.3) |
| ***BzBr bound CYP2C9-1R9O*** | Flurbiprofen | -7.6 | Thr 364; Phe 69, Phe 476 | 20.1 (16.7) |
| ***DBHB bound CYP2C9-1R9O*** | Flurbiprofen | -8.8 | Arg 108 | 4.4 (4.4) |
| ***BzBr bound CYP2C9-1R9O*** | Diclofenac | -6.9 | Phe 69, Trp 212 | 22.3 (16.5) |
| ***DBHB bound CYP2C9-1R9O*** | Diclofenac | -8.3 | Phe 100 | 12.3 (4.7) |
| ***Diclofenac bound CYP2C9-1R9O*** | BzBr | -7.3 | Trp 212 | 18.6 (16.0) |
| ***Diclofenac bound CYP2C9-1R9O*** | DBHB | -5.5 | Arg 108 | 11.6 (11.6) |

**Material B: NMR (^1^H and ^13^C) data and spectra of pure BzBr derivatives synthesized in laboratory.**

**Methylated Benzarone (Figure S5 & S6):** Benzarone (0.93 mg, 3.49 mmol) was used to obtain 0.97 mg of the methylated product after purification with flash chromatography (6% ethyl acetate/hexane). TLC (R*_f_* = 0.28, 10% ethyl acetate/hexane). **^1^H NMR** (400 MHz, CDCl_3_, 25 °C): δ = 7.81 (d, *J* = 8.4 Hz, 2H; *H*_Ar_), 7.44 (d, *J* = 8.4 Hz, 1H; *H*_Ar_), 7.36 (d, *J* = 7.6 Hz, 1H; *H*_Ar_), 7.28 - 7.2 (m, 1H; *H*_Ar_), 7.19 - 7.1 (m, 1H; *H*_Ar_), 6.92 (d, *J* = 8.4 Hz, 2H; *H*_Ar_), 3.86 (s, 3H: OC*H*_3_), 2.88 (q, *J* = 7.6 Hz, 2H; C*H_2_*_(alk)_), 1.30 (t, *J* = 7.6 Hz, 3H; C*H_3_*_(alk)_); **^13^C NMR** (100 MHz, CDCl_3_, 25°C): δ = 190.7, 165.6, 163.6, 153.7, 132.0, 131.8, 127.3, 124.3, 123.5, 121.4, 116.3, 113.8, 111.1, 55.6, 21.9, 12.5; **IR** (thin film): *ν* = 3463 (br), 2985 (s), 2255 (s), 1738 (s), 1455 (s), 1375 (s), 1259 (s), 1048 (m), 915 (s), 734 (s) cm^-1^; **HRMS (ESI^+^):** calcd. for C_18_H_17_O_3_ (MH^+^) 281.1178, found 281.1168.

**Methylated Benzbromarone (Figure S7 & S8):** Benzbromarone (0.87 mg, 2.04 mmol) was used to obtain 0.89 mg of the methylated product after purification with flash chromatography (5% ethyl acetate/hexane). TLC (R*_f_* = 0.35, 10% ethyl acetate/hexane). **^1^H NMR** (400 MHz, CDCl_3_, 25 °C): δ =7.98 (s, 2H; *H*_Ar_), 7.50 (d, *J* = 8.4 Hz, 1H; *H*_Ar_), 7.42 (d, *J* = 7.6 Hz, 1H; *H*_Ar_), 7.35 - 7.28 ( m, 1H; *H*_Ar_), 7.27 - 7.2 (m, 1H; *H*_Ar_), 3.98 (s, 3H: OC*H*_3_), 2.90 (q, *J* = 7.6 Hz, 2H; C*H_2_*_(alk)_), 1.36 (t, *J* = 7.6 Hz, 3H; C*H_3_*_(alk)_); **^13^C NMR** (100 MHz, CDCl_3_, 25°C): δ = 188.2, 167.1, 157.8, 153.8, 137.3, 133.7, 126.5, 124.9, 124.0, 121.2, 118.6, 115.3, 111.3, 61.0, 22.2, 12.4; **IR** (thin film): *ν* = 3435 (br), 3069 (s), 2930 (m), 1650 (s), 1570 (s), 1459 (s), 1371 (w), 1249 (m), 984 (m), 746 (s) cm^-1^; **HRMS (ESI^+^):** calcd. for C_18_H_15_O_3_Br_2_ (MH^+^) 436.9388, found 436.9376.

**Methylated Benziodarone (Figure S9 & S10):** Benziodarone (0.91 mg, 1.76 mmol) was used to obtain 0.93 mg of the methylated product after purification with flash chromatography (5% ethyl acetate/hexane). TLC (R*_f_* = 0.41, 10% ethyl acetate/hexane). **^1^H NMR** (400 MHz, CDCl_3_, 25 °C): δ = 8.22 (s, 2H; *H*_Ar_), 7.50 (d, *J* = 8.0 Hz, 1H; *H*_Ar_), 7.44 (d, *J* = 8.0 Hz, 1H; *H*_Ar_), 7.38 - 7.29 ( m, 1H; *H*_Ar_), 7.28-7.2 (m, 1H; *H*_Ar_), 3.94 (s, 3H: OC*H*_3_), 2.88 (q, *J* = 7.6 Hz, 2H; C*H_2_*_(alk)_), 1.36 (t, *J* = 7.6 Hz, 3H; C*H_3_*_(alk)_); **^13^C NMR** (100 MHz, CDCl_3_, 25°C): δ = 187.9, 167.0, 162.4, 153.8, 140.9, 138.5, 126.6, 124.9, 124.0, 121.2, 115.5, 111.3, 90.6, 61.0, 22.2, 12.4; **IR** (thin film): *ν* = 3432 (br), 2932 (s), 1727 (s), 1640 (s), 1576 (s), 1379 (s), 1245 (m), 1172 (m), 997 (s), 750 (m) cm^-1^; **HRMS (ESI^+^):** calcd. for C_18_H_15_O_3_I_2_ (MH^+^) 532.9111, found 532.9100.

**Figure S5: NMR (^1^H) spectra for MeOBzr**


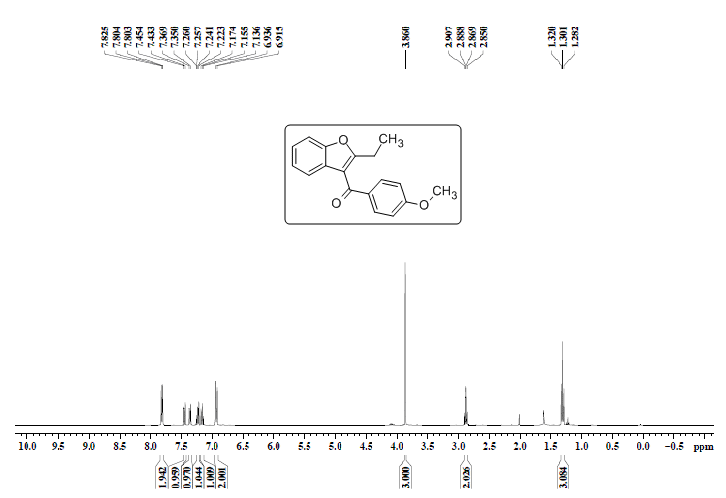


**Figure S6: NMR (^13^C) spectra for MeOBzr**


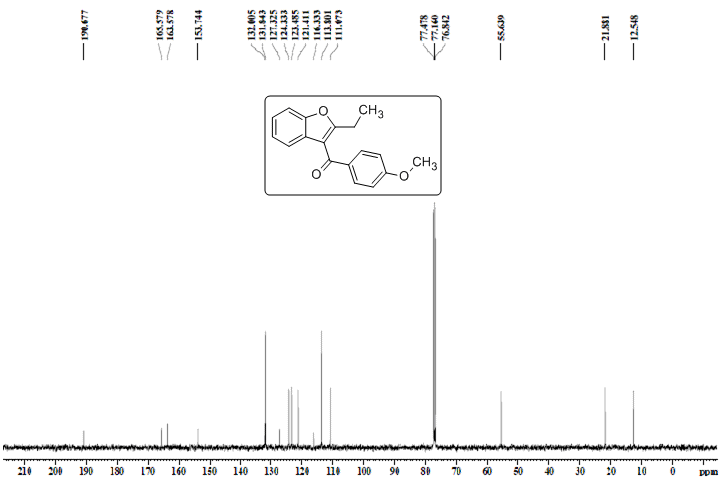


**Figure S7: NMR (^1^H) spectra for MeOBzBr**


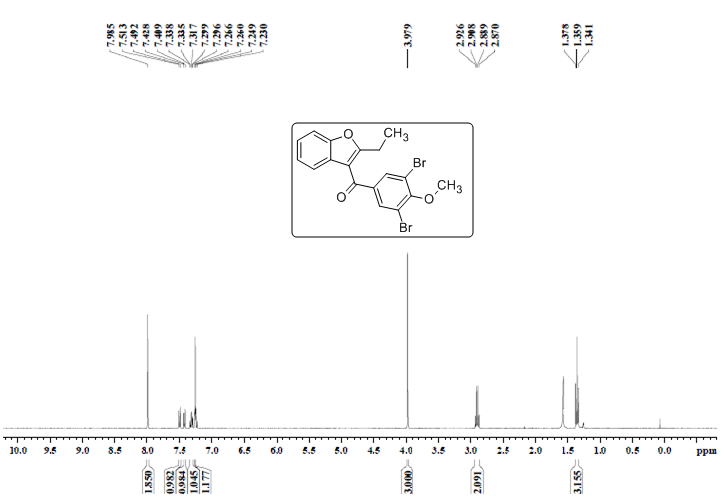


**Figure S8: NMR (^13^C) spectra for MeOBzBr**


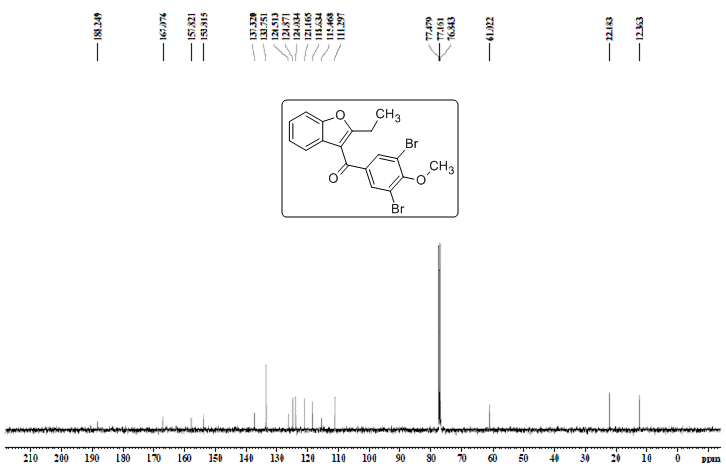


**Figure S9: NMR (^1^H) spectra for MeOBzIr**


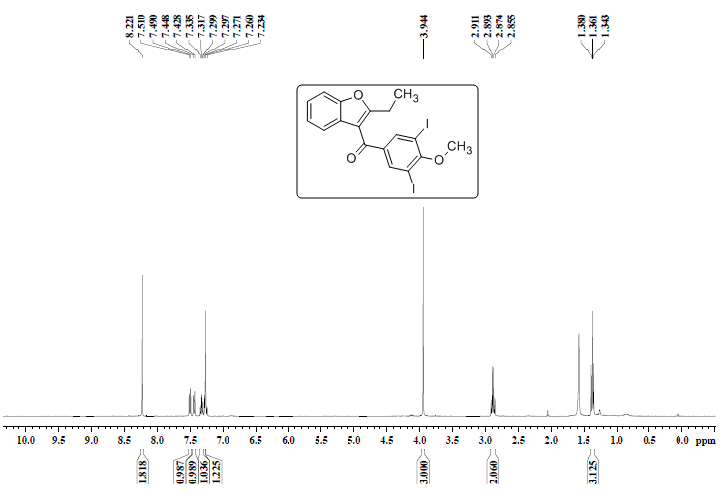


**Figure S10: NMR (^13^C) spectra for MeOBzIr**


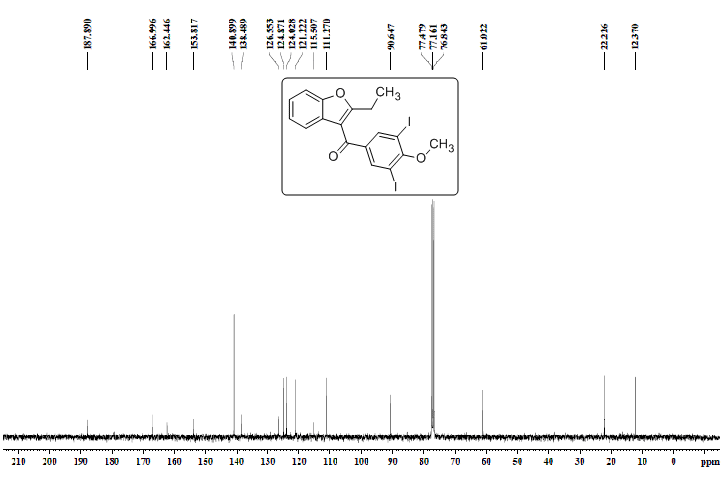

Supplement: File S1 — Figure S1: CYP2C9 (1R9O) – Flurbiprofen. Docked flurbiprofen aligned with the crystal structure of flurbiprofen bound CYP2C9. Figure S2: CYP2C9 (1OG5) – Warfarin. Docked S-warfarin (green) aligned with the crystal structure of flurbiprofen bound CYP2C9. Figure S3: P450cam – Camphor. Aligned camphor bound P450cam crystal structure with the docked camphor. Figure S4: P450BM3- Palmitic acid. Docked palmitic acid represented in alignment with palmitate bound crystal structure of P450BM3. Figure S5: NMR (1H) spectra for MeOBzr. Figure S6: NMR (13C) spectra for MeOBzr. Figure S7: NMR (1H) spectra for MeOBzBr. Figure S8: NMR (13C) spectra for MeOBzBr. Figure S9: NMR (1H) spectra for MeOBzIr. Figure S10: NMR (13C) spectra for MeOBzIr. Table S1: In silico docking analyses of some CYPs (with known crystal structures) for diverse substrates, activators and inhibitors. (DOC) [file pone.0089967.s001.doc]
